# Emergence of high ORR activity through controlling local density-of-states by alloying immiscible Au and Ir†
†Electronic supplementary information (ESI) available. See DOI: 10.1039/c8sc04135k


**DOI:** 10.1039/c8sc04135k

**Published:** 2018-12-04

**Authors:** Kohei Kusada, Dongshuang Wu, Tomokazu Yamamoto, Takaaki Toriyama, Syo Matsumura, Wei Xie, Michihisa Koyama, Shogo Kawaguchi, Yoshiki Kubota, Hiroshi Kitagawa

**Affiliations:** a Division of Chemistry , Graduate School of Science , Kyoto University , Kitashirakawa Oiwake-cho, Sakyo-ku , Kyoto 606-8502 , Japan . Email: kusada@kuchem.kyoto-u.ac.jp ; Email: kitagawa@kuchem.kyoto-u.ac.jp; b Department of Applied Quantum Physics and Nuclear Engineering , Kyushu University , 744 Motooka, Nishi-ku , Fukuoka 819-0395 , Japan; c The Ultramicroscopy Research Center , Kyushu University , 744 Motooka, Nishi-ku , Fukuoka 819-0395 , Japan; d INAMORI Frontier Research Center , Kyushu University , 744 Motooka, Nishi-ku , Fukuoka 819-3095 , Japan; e GREEN , National Institute for Materials Science , 1-1 Namiki , Tsukuba , Ibaraki 305-0044 , Japan . Email: koyama.michihisa@nims.go.jp; f Japan Synchrotron Radiation Research Institute (JASRI) SPring-8 , 1-1-1 Kouto, Sayo-cho, Sayo-gun , Hyogo 679-5198 , Japan; g Department of Physical Science , Graduate School of Science , Osaka Prefecture University , 1-1 Gakuen-cho, Naka-ku, Sakai , Osaka 599-8531 , Japan

## Abstract

The electronic structure of surface atoms has a great effect on catalytic activity because the binding energy of reactants is closely related to the electronic structure. Therefore, designing and controlling the local density of states (LDOS) of the catalyst surface would be a rational way to develop innovative catalysts. Herein, we first demonstrate a highly active AuIr solid-solution alloy electrocatalyst for the oxygen reduction reaction (ORR) by emulating the Pt LDOS profile. The calculated LDOS of Ir atoms on the Au_0.5_Ir_0.5_(111) surface closely resembled that of Pt(111), resulting in suitable oxygen adsorption energy on the alloy surface for the ORR. We successfully synthesized AuIr solid-solution alloys, while Ir and Au are immiscible even above their melting points in the bulk state. Although monometallic Ir or Au is not active for the ORR, the synthesized Au_0.5_Ir_0.5_ alloy demonstrated comparable activity to Pt at 0.9 V *versus* a reversible hydrogen electrode.

## Abstract 2

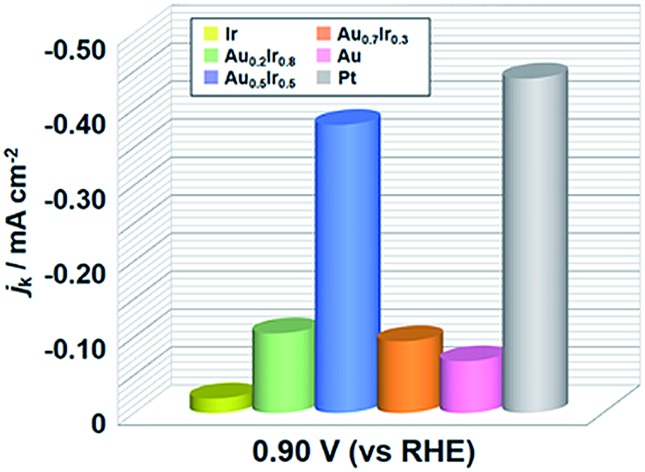
Although Ir or Au is not active for ORR, we first demonstrate highly active Au_0.5_Ir_0.5_ alloy by emulating Pt LDOS profile.

## Introduction

Designing the electronic structure of materials is one of the best ways to tune the material properties because not only physical properties such as magnetic and electrical properties but also chemical properties such as catalytic properties are closely related to the density of states (DOS) of solids.^1^,^2^ In particular, for catalytic reactions, the local DOS (LDOS), which is a projection of DOS to each atom, of the surface atoms is supposed to be more important than the total DOS of a bulk solid because reactants interact with atoms on a solid surface, and the catalytic activity is crucially affected by the binding energy of reactants on the catalyst surface. Therefore, LDOS engineering would realize an innovative catalyst possessing the desirable LDOS profile for targeted reactions. As a demonstration, we aimed to create pseudo-Pt catalysts by emulating the electronic structure of Pt with other elements.

A solid-solution alloy is a favourable material to continuously control its electronic structure. In such an alloy, the constituents randomly mix at the atomic level, and thus, we can continuously control its electronic structure by changing the combination of elements and/or composition of constituents. However, even among d-block elements, the majority of bulk alloy systems are the immiscible type under ambient conditions, hindering us from freely developing solid-solution alloys and designing DOS profiles. Recently, we demonstrated that the nanosize effect offers a chance to find a way out of this metallurgical difficulty; that is, we can obtain metal nanoparticles (NPs) having new phases that do not exist in bulk states.^3^–^6^ In general, a nanoparticle with the same crystal structure as the corresponding bulk material is obtained. However, if we find appropriate synthetic conditions, it allows us to develop novel NPs adopting new phases including solid-solution alloy NPs consisting of immiscible combinations.^6^–^11^ Therefore, by focusing on NPs, we would be able to synthesize any solid-solution alloys regardless of the bulk phase diagrams, leading us to create effective catalysts.

On the basis of LDOS engineering, we have focused on an AuIr solid-solution alloy as one of the candidates for pseudo-Pt because Pt is located between Ir and Au on the periodic table of the elements. However, these elements cannot mix with each other at the atomic level, even above their melting points.^12^ Although several papers have been reported on the Au–Ir system, most of them were on the segregated type, and a pure solid-solution alloy had not been obtained.^13^–^20^.

In this study, we emulate the Pt LDOS profile and create AuIr solid-solution alloy NPs over the whole metallic composition. We demonstrate that the atomic-level alloying can realize a Pt-like electronic structure at the Ir atoms on the Au_0.5_Ir_0.5_(111) surface, and Au_0.5_Ir_0.5_ exhibits comparable oxygen reduction reaction (ORR) activity to that of Pt. To design the Pt-like LDOS and its catalytic properties, we calculated the electronic structure and oxygen binding energy of the alloy using density functional theory (DFT) method. The solid-solution alloy NPs of the bulk-immiscible Au and Ir combination were successfully synthesized by a chemical reduction method. The structure of the materials was characterized by atomic-resolution scanning transmission electron microscopy (STEM) coupled with energy-dispersive X-ray (EDX) spectroscopy and synchrotron X-ray diffraction (XRD). To confirm the emergence of Pt-like property, we examined the ORR activity because the ORR is well-known as the reaction for which Pt shows excellent catalytic activity.

## Results and discussion

### DFT calculations of the electronic structure and oxygen binding energies

**Fig. 1:**
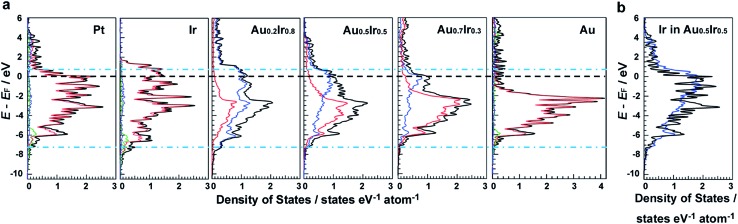
The electronic structures of the surfaces of the catalysts studied by DFT calculations. (a) DOS of the top layer of Pt, Ir, Au and Au_*x*_Ir_1–*x*_(111) surfaces. Blue and red lines in the Au_*x*_Ir_1–*x*_ solid-solution alloy indicate the Ir and Au LDOS, respectively. Red, green and blue lines in the Pt, Ir and Au surfaces show d-, s- and p-bands, respectively. (b) Comparison of the LDOS of the Au_0.5_Ir_0.5_ surface with the LDOS of the Pt surface (blue, Ir-LDOS in the Au_0.5_Ir_0.5_ solid-solution alloy; black, Pt).

DFT calculations were performed to investigate the electronic structures of monometallic Ir, Pt and Au, as well as Au_*x*_Ir_1–*x*_ alloys. Fig. 1a shows the comparison of the electronic structures of the (111) surface, which is the most stable surface of the face-cantered cubic (fcc) structure. It is clearly seen that the electronic structure of Au_*x*_Ir_1–*x*_ is continuously changed from the Ir wider d-band to the Au narrower d-band, and Au_0.5_Ir_0.5_ possesses a unique electronic structure, which is totally different from monometallic Ir or Au. The d-band width of Au_0.5_Ir_0.5_ is most similar to that of Pt, which is shown as blue dotted lines. Pt shows several characteristic DOS peaks and the peaks located near the Fermi energy are relatively high. Although the total DOS profile of the top layer of Au_0.5_Ir_0.5_ is not similar to the Pt profile around the Fermi energy, the LDOS of Ir atoms of the top layer have high DOS near the Fermi energy. Hence, we focused on the LDOS of Ir and Au atoms on the Au_0.5_Ir_0.5_ alloy surface. Fig. 1b and S1 in the ESI† show the LDOS of Ir or Au atoms on the alloy surface as well as the LDOS of Pt atoms on the Pt surface for comparison. It is notable that the LDOS of Ir atoms on the alloy surface is similar to the Pt LDOS profile, whereas Au atoms show a quite different profile. We further investigated the oxygen binding energies on those surfaces to validate if the alloy can exhibit Pt-like ORR activity because ORR is well-known as a reaction in which Pt shows excellent catalytic activity. It is well-known that the binding energy of O is considered to be a significant factor for the ORR activity,^21^ and the ORR activity shows a volcano type dependence on the oxygen binding energy because too weak or too strong of a binding energy cannot promote the ORR.^21^,^22^ Consequently, it is considered that a catalyst having a proper O binding energy can provide high ORR activity. The O binding energies on each surface are summarized in Tables S3–S8.† Compared to monometallic surfaces, there are a variety of adsorption sites on the alloy surface, and we calculated the O binding energies for all possible adsorption sites on the Au_0.5_Ir_0.5_ surface model (Table S6 and Fig. S5†). Since the DOS changes by the atomic orders, we adopted a random alloy surface model to obtain standard adsorption energies on the alloy surface. However, the result shown here is one of the typical examples. The calculated results demonstrated that the adsorption sites involve mainly Ir atoms, which provide binding energies similar to that of Pt. Even if an O atom approaches the sites consisting of only Au atoms, the O atom moves to another site including Ir atoms. Therefore, Ir is considered to play a key role in the O adsorption on the alloy surface. These results show that the Pt-like LDOS profile is realized by the formation of an Au_0.5_Ir_0.5_ solid-solution alloy causing the change in composition and lattice parameter of alloy surface, and this LDOS provides similar O adsorption energies to Pt, suggesting that Au_0.5_Ir_0.5_ shows Pt-like ORR activity.

### Synthesis and characterization

**Fig. 2:**
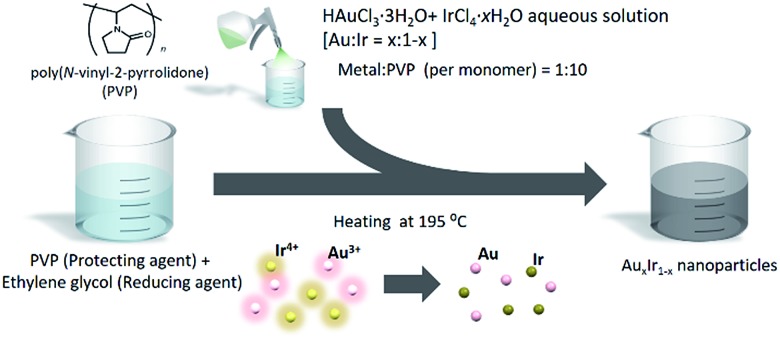
Schematic image of the synthesis of Au_*x*_Ir_1–*x*_ solid-solution alloy NPs.

Due to the immiscible alloy system, synthesizing pure AuIr solid-solution alloy is still challenging even on the nanoscale. To overcome this challenge, we adopted a chemical reduction method that enables us to rapidly reduce different metal ions concurrently under extreme conditions. The method is very simple, as shown in Fig. 2. Both HAuCl_4_·3H_2_O and IrCl_4_·H_2_O were dissolved into deionized water. Meanwhile, poly(*N*-vinyl-2-pyrrolidone) (PVP), a protecting agent, was added to ethylene glycol (EG), a reducing agent, and the solution was heated to 195 °C. The precursor solution was then slowly added to the EG solution, and the metal ions were concurrently reduced in a moment. After the reduction finished, the prepared NPs were separated by centrifugation. On the other hand, if the precursors are dissolved in EG solution before heating, Au ions would be reduced earlier at lower temperature before Ir ions are reduced and then form phase separated NPs due to the difference of reduction potentials and the nature of immiscible Au–Ir system.

The metal composition was controlled by the nominal ratio of the metal precursors, and three types of AuIr NPs were synthesized with different compositions. The metal composition and size of the obtained materials were characterized by X-ray fluorescence (XRF) measurements and transmission electron microscopy (TEM), respectively (Tables S10 and S11 and Fig. S9†). We obtained Au_0.2_Ir_0.8_ (5.5 ± 1.4 nm), Au_0.5_Ir_0.5_ (7.2 ± 1.7 nm) and Au_0.7_Ir_0.3_ (9.9 ± 2.3 nm) NPs and found that the size of the NPs decreased with increasing Ir content under the same synthetic conditions.

**Fig. 3:**
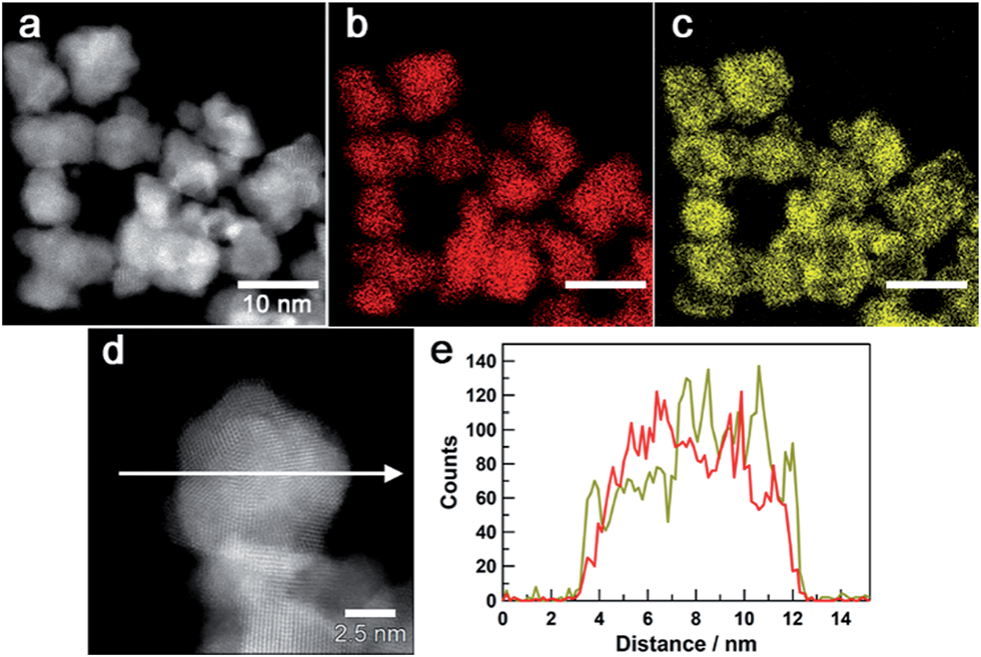
Scanning transmission electron microscopy images of Au_0.5_Ir_0.5_ NPs. (a) a HAADF-STEM image. (b), (c) Au-M and Ir-M STEM-EDX maps (red, Au; yellow, Ir). (e) Compositional line profiles of Au (red) and Ir (dark yellow) for the Au_0.5_Ir_0.5_ nanoparticle recorded along the arrow shown in (d) the HAADF-STEM image.

Fig. 3a–c show a high-angle annular dark-field (HAADF) STEM image of Au_0.5_Ir_0.5_ NPs and the corresponding Au and Ir elemental maps. These results revealed that both Ir and Au atoms were distributed in all of the particles. Furthermore, an atomic resolution HAADF-STEM image is shown in Fig. 3d, and EDX line profiles of Ir-M and Au-M (Fig. 3e) were taken along the white arrow in Fig. 3d. From this result, we found that although the particle was polycrystalline, the two elements were existed in the same crystal grains; thus, the obtained NPs formed a solid-solution structure. The metal composition of the surface of Au_0.5_Ir_0.5_ NP was further investigated by EDX point analysis (Fig. S10†). The average composition *x* in Au_*x*_Ir_1–*x*_ was calculated to be 0.43 ± 0.13. Fig. S11 and S12† show that the obtained Au_0.2_Ir_0.8_ and Au_0.7_Ir_0.3_ also form solid-solution structures.

**Fig. 4:**
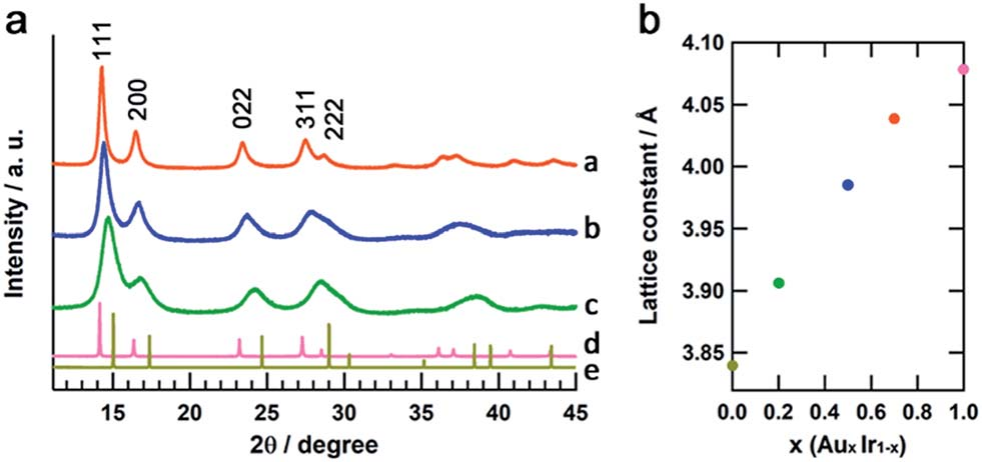
X-ray diffraction analysis of Au_*x*_Ir_1–*x*_ NPs. (a) Powder XRD patterns of a; Au_0.7_Ir_0.3_ NPs, b; Au_0.5_Ir_0.5_ NPs, c; Au_0.2_Ir_0.8_ NPs, d; Au and e; Ir powders. (b) A plot of the changes in the lattice constants verses the metal composition.

The crystal structures of the obtained Au_*x*_Ir_1–*x*_ NPs were confirmed by synchrotron XRD analysis at the beamline BL02B2, SPring-8.^23^Fig. 4a shows the XRD patterns of Au_*x*_Ir_1–*x*_ NPs, the Ir and Au bulk powders. All of the Au_*x*_Ir_1–*x*_ NPs, as well as the Ir and Au metals, show a single fcc pattern, and the diffraction peaks of Au_*x*_Ir_1–*x*_ NPs continuously shifted to lower angles with increasing Au content. Fig. 4b shows the lattice constants of Au_*x*_Ir_1–*x*_ NPs, as determined by LeBail refinement of the XRD patterns. The lattice constants linearly increased with increasing Au content, and this linear correlation between the metal composition and the lattice constant follows Vegard's law,^24^ a well-known empirical rule for solid-solution materials. These results support that we first succeeded in synthesizing pure solid-solution AuIr alloy over the entire composition range.

### Catalytic properties

**Fig. 5:**
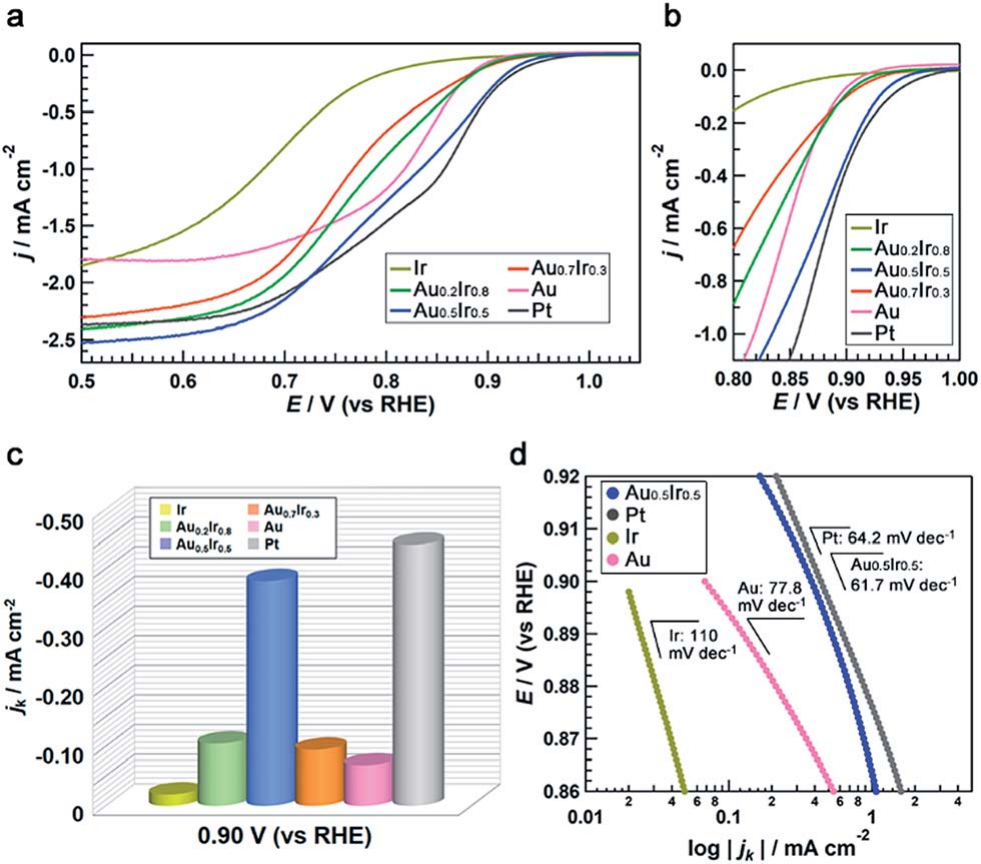
Electrochemical oxygen-reduction activities of Au_*x*_Ir_1–*x*_ alloy catalysts. (a), (b) rotating disk electrode *I*–*V* polarization curves taken in O_2_-saturated 1.0 M NaOH aqueous solution normalized by the glassy carbon electrode geometric area (0.196 cm^2^) (a) from 0.50 to 1.05 V_RHE_ and (b) from 0.80 to 1.00 V_RHE_. (c) Summary of the kinetic current densities for oxygen reduction on each catalyst measured at 0.90 V_RHE_. (d) Tafel plots for the catalysts. All of the NPs were loaded on carbon support.

To demonstrate the emergence of Pt-like property, we investigated the ORR activity of the AuIr alloy NPs as a probe reaction. We also synthesized monometallic Ir, Pt and Au NPs by chemical reduction methods to compare the ORR activity (see the ESI†). These monometallic NPs were prepared to have similar average diameters to that of Au_0.5_Ir_0.5_ to avoid the size effect, but we were not able to control the size of Ir NPs. All of the obtained NPs were supported on carbon black (Vulcan XC-72R), and the amount of metal in the catalysts was fixed at 20 wt%. Measurements were performed with a rotating disk electrode (RDE) at a scanning speed of 5 mV s^–1^ in 1.0 M NaOH aqueous solution. The current density was normalized by the glassy carbon electrode geometric area (0.196 cm^2^). The polarization curves in Fig. 5a and b show that Pt catalyzes the reaction at a much lower overpotential compared to the Ir and Au catalysts, as is the case in previous reports.^25^,^26^ It is notable that the Au_0.5_Ir_0.5_ alloy begins to catalyze the reaction at approximately 1.0 V *versus* the reversible hydrogen electrode (RHE), which is comparable to the onset potential of the Pt catalyst. Fig. 5c shows the comparison of the kinetic current density (*j*_k_) on each catalyst measured at 0.9 V_RHE_. The kinetic current was calculated based on the Koutecky–Levich equation as follows:1/*i* = 1/*i*_k_ + 1/*i*_d_where *i*, *i*_k_, and *i*_d_ are the measured current, kinetic current and diffusion-limiting current, respectively. Au_0.5_Ir_0.5_ shows a comparable kinetic current density to that of Pt at this potential. Furthermore, the Tafel slopes of the Pt, Au_0.5_Ir_0.5_, Au and Ir catalysts were 64.2, 61.7, 77.8 and 110 mV dec^–1^, respectively (Fig. 5d). The slope of Au_0.5_Ir_0.5_ is smaller than those of Au and Ir; this slope suggests the improvement of the kinetics for the reaction. Interestingly, the similarity of the slopes of Pt and Au_0.5_Ir_0.5_ implies that the reaction mechanism of these catalysts could be similar. From these results, we found that although Ir and Au catalysts do not show good activity for this reaction, the Au_0.5_Ir_0.5_ catalyst showed higher activity, which was comparable to that of Pt. This result is regarded as an example revealing the possibility of LDOS engineering as a rational strategy to design effective catalysts.

The same catalytic tests in 0.05 M H_2_SO_4_ aqueous solution were also performed (Fig. S14†). It was a little different from the alkaline case, ORR activity of Au_0.5_Ir_0.5_ was not comparable to that of Pt. However, the activity of Ir and Au was much worse than the alloy and the tendency was the same as the alkaline case. This might be caused by the nature that only Pt can exhibit high activity in both acidic and alkaline solutions, while Ir and Au show pretty low activity in acidic condition. Furthermore, we performed an accelerated durability test (ADT) to investigate the catalytic stability of Au_0.5_Ir_0.5_ catalyst by cyclic potential sweeps between 0.65 and 0.95 V (*vs.* RHE) at 100 mV s^–1^ in O_2_-saturated 1.0 M NaOH aqueous solution at room temperature. The integral of the hydrogen under potential deposition (H_upd_) region between 0.1 to 0.4 V (*vs.* RHE) was compared (Table S13†). After 10 000 cycles, the CV measurement showed a loss of only 7.9% in the H_upd_ peak and the catalyst still showed better activity for ORR than monometallic Ir or Au catalyst (Fig. S15†).

## Conclusions

In summary, we successfully synthesized AuIr solid-solution alloy NPs over the entire composition range by a chemical reduction method, although Au and Ir are immiscible even in a high-temperature liquid phase in the bulk phase diagram. We demonstrated that Ir atoms on the Au_0.5_Ir_0.5_ alloy surface possess a Pt-like LDOS, and therefore, the alloy catalyst shows an excellent ORR activity comparable to that of the Pt catalyst, even though monometallic Ir or Au catalyst shows poor activity. This work represented untapped potentials of novel solid-solution alloy NPs of immiscible systems and proved the usefulness of LDOS engineering which is a rational strategy for designing and developing desirable catalysts.

## Conflicts of interest

There are no conflicts to declare.

## Supplementary Material

Supplementary information
