# Supplementary material for: Emergence of high ORR activity through controlling local density-of-states by alloying immiscible Au and Ir
Source: Chem Sci. 2018 Dec 4;10(3):652–6. doi: 10.1039/c8sc04135k (PMC6349063; doi:10.1039/c8sc04135k)
Supplement: Supplementary file 1 [file SC-010-C8SC04135K-s001.pdf]

Electronic Supplementary Material (ESI) for Chemical Science.
This journal is © The Royal Society of Chemistry 2018

Supporting Information

Emergence of High ORR activity through Controlling Local Density-of-States by Alloying Immiscible Au and Ir

Kohei Kusada[1*], Dongshuang Wu[1], Tomokazu Yamamoto[2], Takaaki Toriyama[3], Syo Matsumura[2, 3, 4], Wei Xie[4], Michihisa Koyama[4, 5*], Shogo Kawaguchi[6], Yoshiki Kubota[7] and Hiroshi Kitagawa[1, 4 *]

[1]*Division of Chemistry, Graduate School of Science, Kyoto University, Kitashirakawa Oiwake-cho, Sakyo-ku, Kyoto 606-8502, Japan*

[2]*Department of Applied Quantum Physics and Nuclear Engineering, Kyushu University, 744 Motooka, Nishi-ku, Fukuoka 819-0395, Japan*

[3]*The Ultramicroscopy Research Center, Kyushu University, 744 Motooka, Nishi-ku, Fukuoka 819-0395, Japan*

[4]*INAMORI Frontier Research Center, Kyushu University, 744 Motooka, Nishi-ku, Fukuoka 819-3095, Japan*

[5]*GREEN, National Institute for Materials Science, 1-1 Namiki, Tsukuba, Ibaraki 305-0044, Japan*

[6]*Japan Synchrotron Radiation Research Institute (JASRI) SPring-8, 1-1-1 Kouto, Sayo-cho, Sayo-gun, Hyogo 679-5198, Japan*

[7]*Department of Physical Science, Graduate School of Science, Osaka Prefecture University, 1-1 Gakuen-cho, Naka-ku, Sakai, Osaka 599-8531, Japan*

**These authors contributed equally to this work*

1. Methods

Theoretical methods. All of the spin polarized calculations were carried out on the Vienna ab initio simulation package (VASP),[1,2] version 5.3.3, which is a plane wave density functional code. The electron-electron exchange and correlation interactions were described by using the generalized gradient approximation (GGA) with the form of the Perdew-Burke-Ernzerhof (PBE)[3] functional. The projector augmented wave (PAW)[4,5] method was employed to describe the interactions between the core and valence electrons. The wave functions were expanded in plane wave basis sets with a cut-off energy of 400 eV. The convergence criteria for the electronic self-consistent iteration and the ionic relaxation loop were set at values of 1×10^{-5} eV and 1×10^{-4} eV/Å, respectively. Brillouin zone integrations were performed with k-point sampling using the Monkhorst-Pack (MP)[6] of 9 × 9 × 9 and 7 × 7 × 1 for the bulk and slab models, respectively. The method of Methfessel-Paxton was employed to determine the valence electron occupancies with a smearing width of 0.1 eV.

The slab models of Pt(111), Ir(111), Au(111), and Au-Ir(111) were constructed with four layers and 4 × 4 surface unit cells. The atoms in the bottom two layers were fixed while the rest of atoms were fully relaxed. We employed the Warren-Cowley (WC) parameter[7] to evaluate the randomness of every slab.

The binding energy (E_B) is defined as:

$$E_B = E_{O-slab} - E_{slab} - E_O$$

where the term E_{O-slab} is the total energy for O adsorbed structures, E_{slab} is the total energy for the metal slab structures, and E_O is the energy of an isolated O atom.

Characterization. The atomic ratios of Au and Ir in the NPs were determined by a Rigaku ZSX Primus IV XRF instrument. TEM images were taken using a Hitachi

HT7700 operated at 100 kV. HAADF–STEM and EDX analyses were recorded on a JEM-ARM 200CF STEM instrument operated at 200 kV. Synchrotron XRD patterns were measured at the BL02B2 beamline, SPring-8. The radiation wavelength was 0.5797 Å, and the scan step was 0.006°.

Catalyst preparation. The as-prepared Au_xIr_{1-x}, Ir, Pt and Au NPs were loaded on carbon powder (Vulcan XC-72R, Cabot) by sonicating the powder mixtures in 1:4 deionized water and 2-propanol for 4 h. The amount of metal in the catalysts was fixed at 20 wt%. After collecting the carbon-loaded NP powder by the centrifugation and drying process, catalysts inks were prepared by sonicating the mixture of the catalyst powder (15.0 mg), deionized water (0.6 ml), 2-propanol (2.25 ml) and Nafion solution (0.15 ml, 5% Nafion™ Dispersion Solution DE521 CS type, Wako) for 30 min. The concentration of metal in the inks was 1.0 mg/ml. A 4.0 µl aliquot of the catalyst ink was pipetted onto a polished glassy carbon surface of an RDE and dried in air to form a homogeneous thin film.

Electrochemical testing. All electrochemical tests were performed using a CHI 760e electrochemical workstation (CHI instruments, Inc., USA) connected with a RRDE-3A constant rotation system (ALS Co., Ltd, Japan). We used a conventional three electrode system: a Hg/HgO reference electrode (1.0 M NaOH), a Pt coiled wire counter electrode (57 mm in length) and an RDE (5 mm disk diameter) as a working electrode. The reference electrode was calibrated at r.t. before each test, and all potentials are reported relative to the RHE. All of the catalysts had the same metal mass weight and were loaded onto a glassy carbon RDE electrode. Each catalyst was swept from 0.04 to 1.24 V at a sweep rate of 200 mV/s for several hundreds of cycles in an Ar-saturated 1.0 M NaOH solution to obtain a stable and clean electrode surface before the ORR tests. After the electrochemical cleaning, cyclic voltammetry (Figure S13) was measured at a sweep rate of 20 mV/s. To determine the ORR activity, linear sweep voltammetry

(LSV) was measured at a sweep rate of 5 mV/s at 1600 rpm in an O_2-saturated 1.0 M NaOH solution. All polarization curves were corrected for the *i*R drop in the solution after each scan. All of the electrochemical measurements were repeated at least 3 times to confirm the reproducibility.

(1) Kresse; Furthmüller. Efficient Iterative Schemes for Ab Initio Total-Energy Calculations Using a Plane-Wave Basis Set. *Phys. Rev. B. Condens. Matter* **1996**, *54*, 11169–11186.

(2) Kresse, G. Ab Initio Molecular Dynamics for Liquid Metals. *Phys. Rev. B* **1995**, *47*, 558–561.

(3) Perdew, J. P.; Burke, K.; Ernzerhof, M. Generalized Gradient Approximation Made Simple. *Phys. Rev. Lett.* **1996**, *77*, 3865–3868.

(4) Kresse, G.; Joubert, D. From Ultrasoft Pseudopotentials to the Projector Augmented-Wave Method. *Phys. Rev. B* **1999**, *59*, 1758–1775.

(5) Blöchl, P. E. Projector Augmented-Wave Method. *Phys. Rev. B* **1994**, *50*, 17953–17979.

(6) Hendrik J. Monkhorst, J. D. P. Special Points for Brillouin-Zone Integrations. *Phys. Rev. B* **1976**, *13*, 5188–5192.

(7) Cowley, J. M. An Approximate Theory of Order in Alloys. *Phys. Rev.* **1950**, *77*, 669–675.

2. The comparison of the LDOS of Ir or Au atoms on the $Au_{0.5}Ir_{0.5}(111)$ surface with the Pt(111) surface

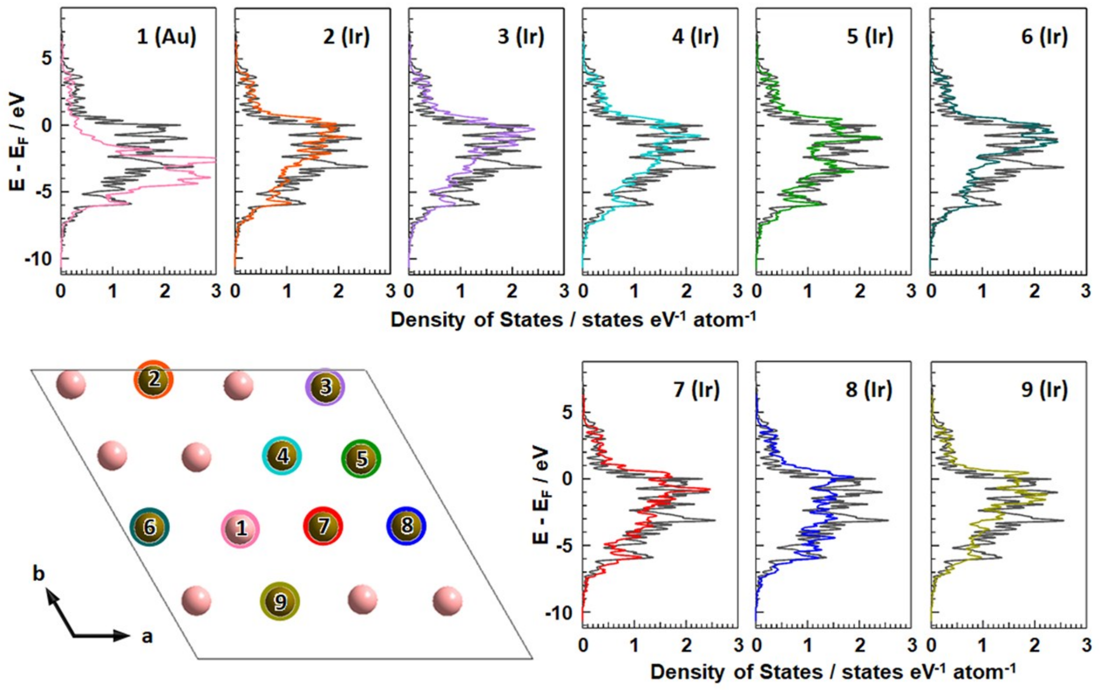

Figure S1 The comparison of the LDOS of $Au_{0.5}Ir_{0.5}(111)$ and Pt(111) surfaces. The Pt LDOS is shown as grey colour and the Au or Ir LDOS is shown as the other colours. The number indicates the position of the Au or Ir atom on the $Au_{0.5}Ir_{0.5}(111)$ surface shown in the lower left. This layer is the top layer of the 4-layer slab model of the $Au_{0.5}Ir_{0.5}(111)$ surface shown in Figure S5. Dark yellow and pink spheres indicate Ir and Au atoms, respectively. The details of the structural model are explained in Figure S5.

Table S1. *d*-band centre of Ir LDOS in of $Au_{0.5}Ir_{0.5}(111)$ shown in Figure S1

Ir site	2	3	4	5	6	7	8	9	Ave.
d-band centre (eV)	-2.78	-2.66	-2.88	-3.00	-2.59	-2.88	-3.18	-2.84	-2.85

Table S2. *d*-band centre and integral of total and local DOS

	d-band center (eV)			Integral (states atom^{-1})		
	TDOS	Ir LDOS	Au LDOS	TDOS	Ir LDOS	Au LDOS
Ir	-3.34			8.16		
$Au_{0.2}Ir_{0.8}$	-3.27	-3.19	-3.49	8.48	6.08	2.40
$Au_{0.5}Ir_{0.5}$	-3.24	-2.85	-3.57	8.81	4.01	4.80
$Au_{0.7}Ir_{0.3}$	-3.24	-2.68	-3.45	9.08	2.50	6.58
Au	-3.53			9.56		
Pt	-2.87			9.1		

3. O binding energies on Ir, Pt, Au and $Au_{0.5}Ir_{0.5}$(111) surfaces

3. 1. Ir(111) surface

Table S3. Oxygen adsorption site and binding energy on the Ir(111) surface

	Site	Binding energy (eV)
1	Top	-3.97
2	Bridge (→ fcc)	-5.04
3	fcc	-5.04
4	hcp	-4.82

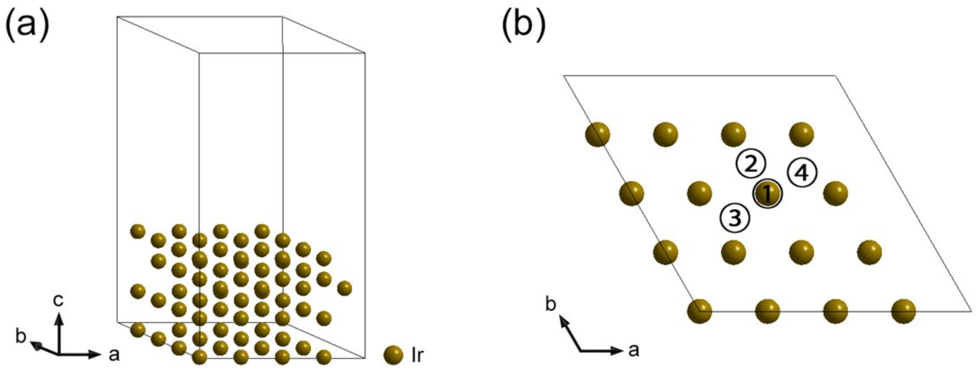

Figure S2 (a) Side view of the 4-layer slab model of the Ir(111) surface. (b) Top view of the top layer. The numbers in (b) the top layer indicate the adsorption sites listed in Table S3. The calculated binding energies on the monometallic Ir surface are similar to those found in previous reports[8].

(8) Krekelberg, W. P., Greeley, J. & Mavrikakis, M. Atomic and Molecular Adsorption on Ir(111). *J. Phys. Chem. B* **2004**, *108*, 987–994.

3. 2. Pt(111) surface

Table S4. Oxygen adsorption site and binding energy on the Pt(111) surface

	Site	Binding energy (eV)
1	Top	-2.97
2	Bridge (→ fcc)	-4.30
3	fcc	-4.30
4	hcp	-3.81

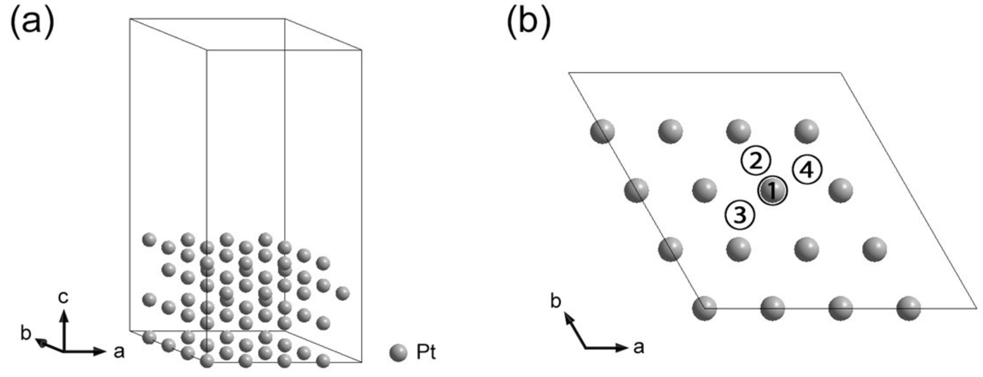

Figure S3 (a) Side view of the 4-layer slab model of the Pt(111) surface. (b) Top view of the top layer. The numbers in (b) the top layer indicate the adsorption sites listed in Table S4. The calculated binding energies on the monometallic Pt surface are similar to those found in previous reports[9].

(9) Li, R., Li, H. & Liu, J. First principles study of O2 dissociation on Pt(111) surface: Stepwise mechanism. *Int. J. Quantum Chem.* **2016**, *116*, 908–914.

3. 3. Au(111) surface

Table S5. Oxygen adsorption site and binding energy on the Au(111) surface

	**Site**	**Binding energy (eV)**
1	1 Top	N/A
2	2 Bridge (→ fcc)	-3.01
3	3 fcc	-3.01
4	4 hcp	-2.87

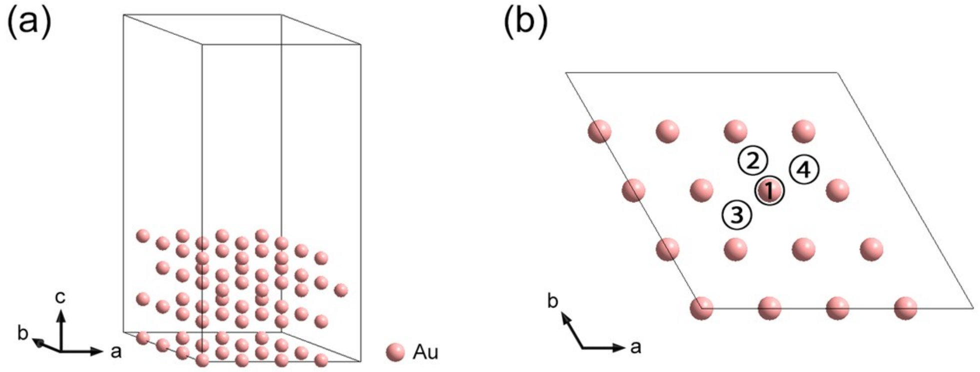

Figure S4 (a) Side view of the 4-layer slab model of the Au(111) surface. (b) Top view of the top layer. The numbers in (b) the top layer indicate the adsorption sites listed in Table S5. The calculated binding energies on the monometallic Au surface are similar to those found in previous reports[10].

(10) Okazaki-Maeda, K. & Kohyama, M. Atomic oxygen adsorption on Au(100) and Au(111): Effects of coverage. *Chem. Phys. Lett.* **2010**, *492*, 266–271.

3. 4. $Au_{0.5}Ir_{0.5}(111)$ surface

Table S6. Oxygen adsorption site and binding energy on the $Au_{0.5}Ir_{0.5}(111)$ surface

	**Site**	**Binding energy (eV)**
1	Ir top	-4.30
2	Au top	N/A
3	Ir-Ir bridge	-4.68
4	Au-Au bridge	N/A
5	Ir-Au bridge (→Ir top like)	-4.26
6	Ir_3 fcc	-4.78
7	Ir_2Au_1 fcc (→Ir-Ir bridge)	-4.68
8	Ir_1Au_2 fcc (→Ir top like)	-4.20
9	Au_3 fcc	-3.20
10	Ir_3 hcp	-4.76
11	Ir_2Au_1 hcp (→Ir-Ir bridge)	-4.68
12	Ir_1Au_2 hcp (→Ir top like)	-4.20
13	Au_3 hcp	N/A

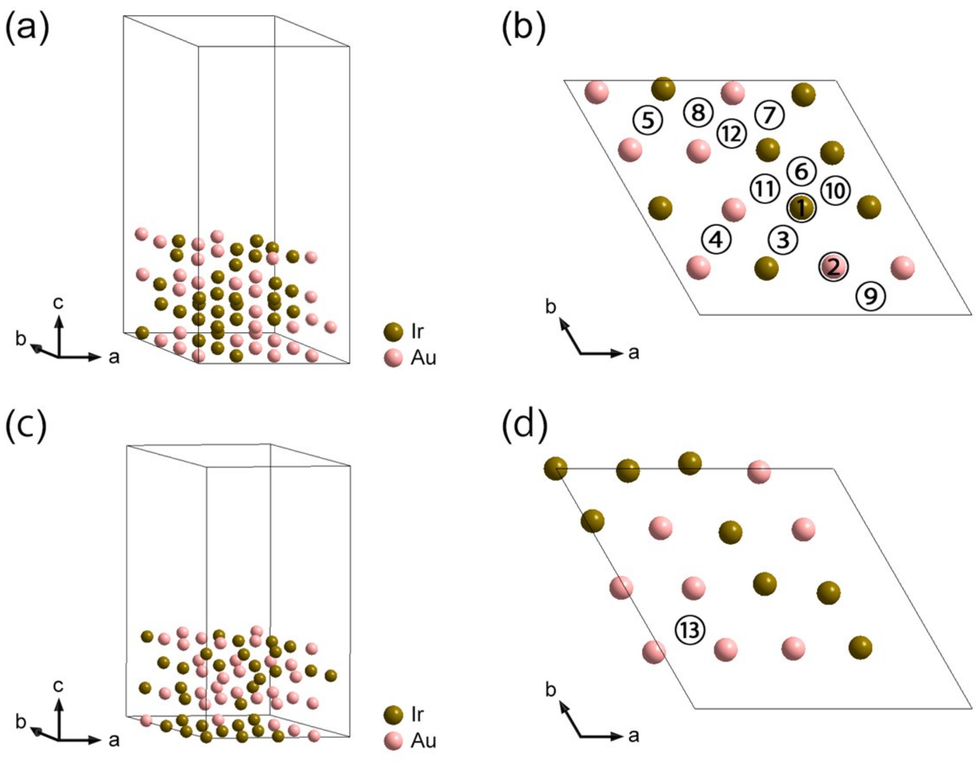

Figure S5 (a) and (c) Side views of the 4-layer slab models of the $Au_{0.5}Ir_{0.5}(111)$ surface. (b) and (d) Top view of the top layers. The numbers in (b) the top layer indicate the adsorption sites listed in Table S6.

The alloy slab model was chosen as an ideal solid-solution structure given the Warren-Cowley (WC) parameter of 0[7]. Since the above model does not have an Au_3-hcp site, the binding energy of the Au_3-hcp site was obtained using another surface model giving the WC parameter of 0 (Figures 5c and d); this model also provides similar energies at other sites. The WC parameter (a_i) was calculated according to the following formula:

$$a_i = 1 - \frac{P_A^i}{C_A}$$

For a binary A–B alloy system, where P_A^i is the conditional probabilities of having B atoms as neighbours in the *i*-th coordination sphere of the substrate, C_A is the concentration of atoms. If a_i equals 0, it indicates that the alloy structure is complete randomness. If $a_i < 0$, it indicates that the A–B bonds are dominant in the alloy system

(local order or supercell), while if α_i> 0, it indicates that A–A bonds are dominant in the alloy system (phase-separated or clustered). Therefore, if the model has WC parameters with a value of 0, the constructed slab is a solid-solution structure.

3. 5. $Au_{0.7}Ir_{0.3}$(111) surface

Table S7. Oxygen adsorption site and binding energy on the $Au_{0.7}Ir_{0.3}$(111) surface

	Site	Binding energy (eV)
1	Ir top	-4.54
2	Au top	N/A
3	Ir_3 fcc	-5.00
4	Ir_2Au_1 fcc (→Ir-Ir bridge)	-4.80
5	Ir_1Au_2 fcc (→Ir top like)	-4.47
6	Au_3 fcc	-3.35
7	Ir_3 hcp	-4.64
8	Ir_2Au_1 hcp (→Ir-Ir bridge)	-4.82
9	Ir_1Au_2 hcp (→Ir top like)	–4.49
10	Au_3 hcp	N/A

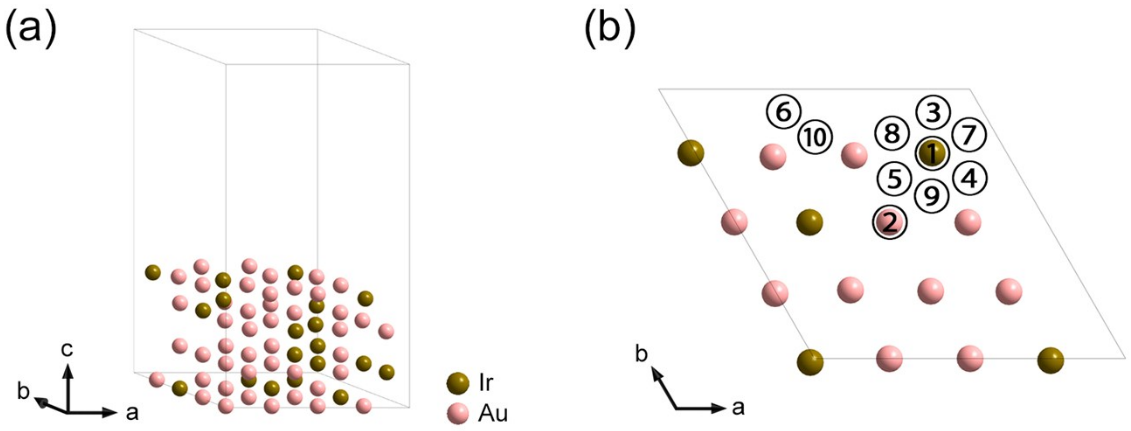

Figure S6 (a) and (c) Side views of the 4-layer slab models of the $Au_{0.7}Ir_{0.3}(111)$ surface. (b) and (d) Top view of the top layers. The numbers in (b) the top layer indicate the adsorption sites listed in Table S7.

The alloy slab model was chosen as an ideal solid-solution structure given the Warren-Cowley (WC) parameter of 0[7].

3. 6. $Au_{0.2}Ir_{0.8}(111)$ surface

Table S8. Oxygen adsorption site and binding energy on the $Au_{0.2}Ir_{0.8}(111)$ surface

	**Site**	**Binding energy (eV)**
1	Ir top	-4.12
2	Au top	N/A
3	Ir_3 fcc	-5.05
4	Ir_2Au_1 fcc (→Ir-Ir bridge)	N/A
5	Ir_1Au_2 fcc (→Ir top like)	N/A

6	Au_3 fcc	-3.55
7	Ir_3 hcp	-5.14
8	Ir_2Au_1 hcp (→Ir-Ir bridge)	N/A
9	Ir_1Au_2 hcp (→Ir top like)	-4.18
10	Au_3 hcp	-3.72

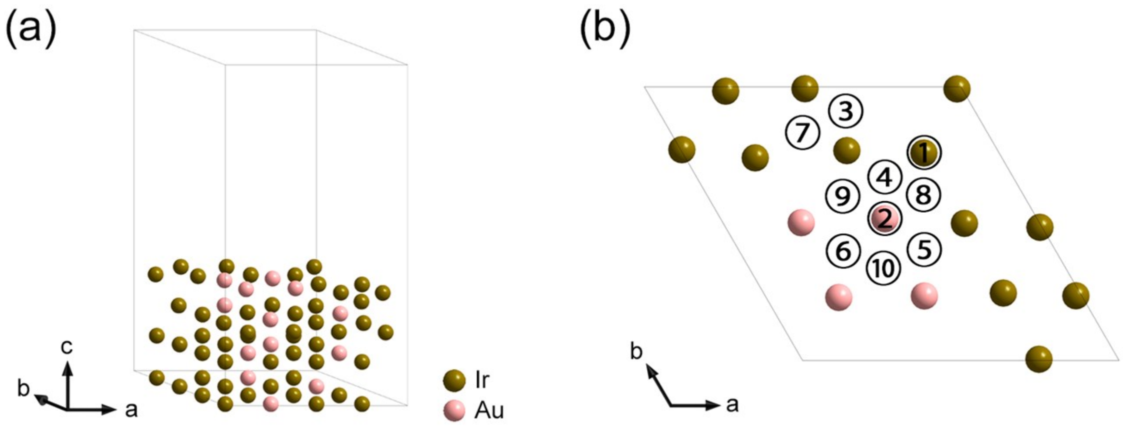

Figure S7 (a) and (c) Side views of the 4-layer slab models of the $Au_{0.2}Ir_{0.8}(111)$ surface. (b) and (d) Top view of the top layers. The numbers in (b) the top layer indicate the adsorption sites listed in Table S8.

The alloy slab model was chosen as an ideal solid-solution structure given the Warren-Cowley (WC) parameter of 0[7].

In all of the alloy surfaces, although the adsorption energy at Ir top site can be discussed by simple d-band centre theory, the tendency of the adsorption property at other sites such as hollow sites cannot be easily explained. In order to explain the tendency of the adsorption property, we need to consider other descriptors to be discussed elsewhere.

4. Synthesis details

Au_xIr_{1-x}NPs synthesis. In a typical synthesis of Au_xIr_{1-x} NPs (x = 0.5), poly(*N*-vinyl-2-pyrrolidone) (PVP, 668.9 mg, MW ≈ 40000, Wako) was dissolved in ethylene glycol (EG, 300 ml, Wako), and the solution was heated to 195 °C with magnetic stirring. Meanwhile, $HAuCl_4$ (94.6 mg, Aldrich) and $IrCl_4$•xH_2O (133.5 mg, Mitsuwa Chemicals) were dissolved in deionized water (40 ml). The precursor solution was then slowly added to the EG solution. The solution was maintained at 195 °C while adding the solution. After cooling to room temperature, the prepared NPs were separated by centrifugation. Other Au_xIr_{1-x} (x = 0.2 and 0.7) NPs were prepared by controlling the molar ratio of Au^{3+} and Ir^{4+} ions. The details of the synthetic conditions for Au_xIr_{1-x} NPs are summarized in Table S9.

Table S9. The synthetic conditions for Au_xIr_{1-x} NPs

	$HAuCl_4$	$IrCl_4$•xH_2O	PVP
$Au_{0.2}Ir_{0.8}$	39.5 mg	166.4 mg	664.3 mg
$Au_{0.5}Ir_{0.5}$	94.6 mg	133.5 mg	668.9 mg
$Au_{0.7}Ir_{0.3}$	165.5 mg	66.6 mg	672.6 mg

Ir NP synthesis. Poly(N-vinyl-2-pyrrolidone) (PVP, 55.5 mg, MW ≈ 40000, Wako) was dissolved in water (25 ml), and the solution was heated to 100 °C with magnetic stirring. Meanwhile, $IrCl_4$•xH_2O (37.0 mg, Mitsuwa Chemicals) and $NaBH_4$ (56.5 mg, Wako) were dissolved in deionized water (5 ml) and EtOH (5 ml, Wako), respectively. The precursor and $NaBH_4$ solutions were then alternately dropped into the PVP

solution. The solution was maintained at 100 °C while adding the solution. After cooling to room temperature, the prepared NPs were separated by centrifugation.

Pt NP synthesis. Poly(N-vinyl-2-pyrrolidone) (PVP, 111.1 mg, MW ≈ 40000, Wako) was dissolved in EG (100 ml, Wako), and the solution was heated to 170 °C with magnetic stirring. Meanwhile, K_2PtCl_4 (332.1 mg, Aldrich) was dissolved in deionized water (20 ml). The precursor solution was then slowly added to the EG solution. The solution was maintained at 170 °C while adding the solution. After cooling to room temperature, the prepared NPs were separated by centrifugation.

Au NP synthesis. Poly(N-vinyl-2-pyrrolidone) (PVP, 555.5 mg, MW ≈ 40000, Wako) and $HAuCl_4$ (39.4 mg, Aldrich) were dissolved in EG (60 ml, Wako), and the solution was stirred at RT. Meanwhile, NaOH (199.9 mg, Wako) was dissolved in deionized water (2 ml). The NaOH solution was then added to the EG solution. The solution was stirred for 30 min at RT. After the reaction was over, the prepared NPs were separated by centrifugation.

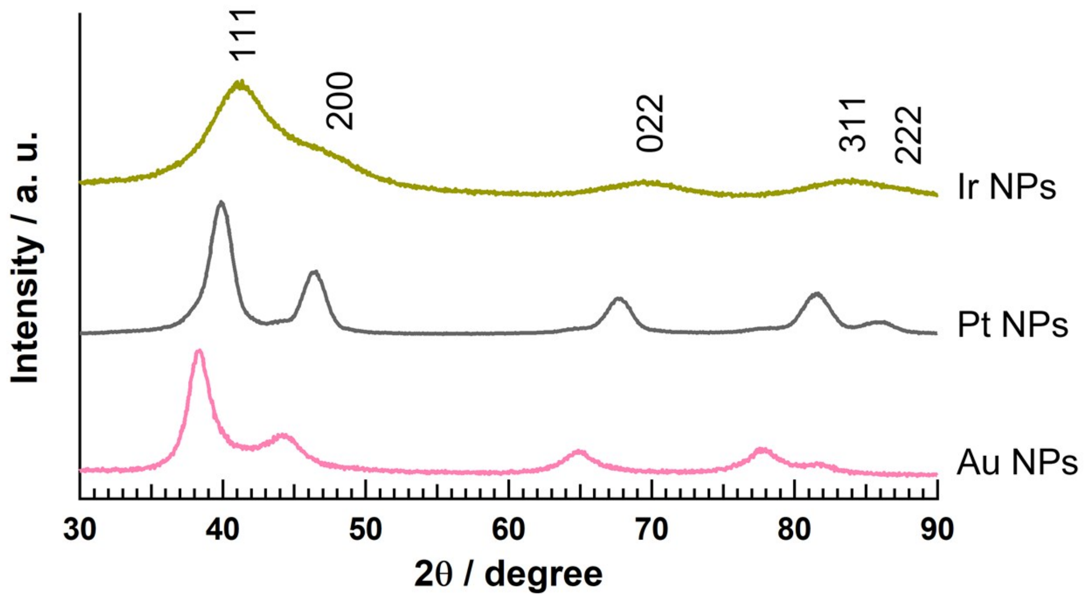

Figure S8 XRD patterns of the synthesized Au, Ir and Pt NPs.

5. Elemental analysis of the prepared Au_xIr_{1-x} NPs

The metal composition of the obtained NPs was characterized by X-ray fluorescence measurements using a Rigaku ZSX Primus IV XRF instrument. The average value was calculated from three measurements, as shown in Table S10.

Table S10. The atomic ratio of Au and Ir in Au_xIr_{1-x} NPs

	Au (at%)	Ir (at%)
$Au_{0.2}Ir_{0.8}$	22.9	77.1
$Au_{0.5}Ir_{0.5}$	50.5	49.5
$Au_{0.7}Ir_{0.3}$	70.0	30.0

6. TEM images and the average size of the prepared NPs

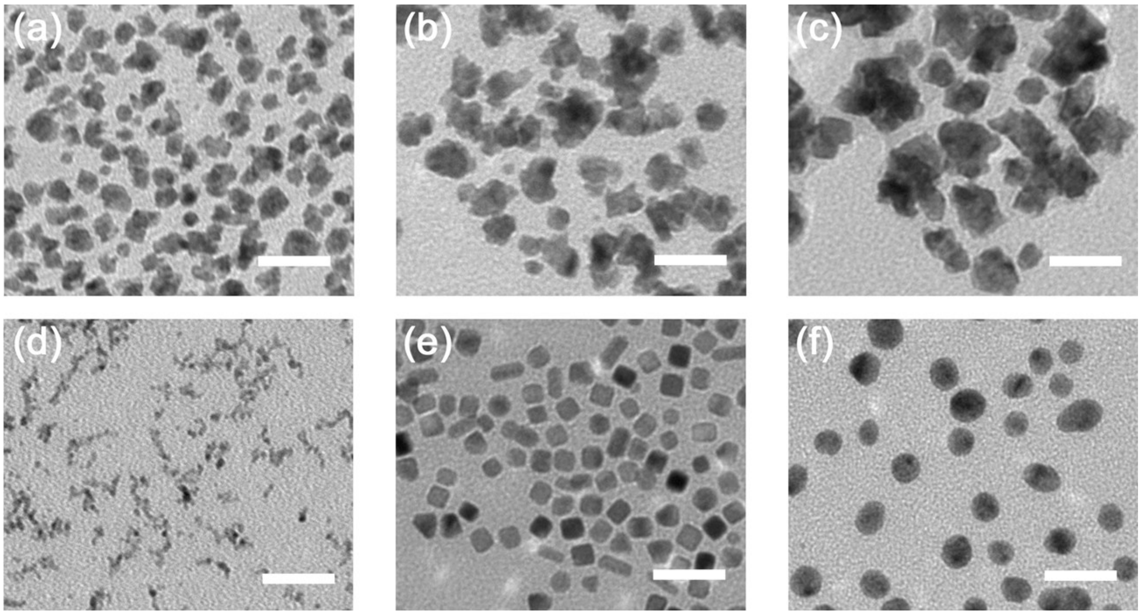

Figure S9 TEM images of (a) $Au_{0.2}Ir_{0.8}$, (b) $Au_{0.5}Ir_{0.5}$, (c) $Au_{0.7}Ir_{0.3}$, (d) Ir, (e) Pt, and (f) Au NPs. The scale bars are 20 nm.

Table S11. The average size of the obtained NPs

	$Au_{0.2}Ir_{0.8}$	$Au_{0.5}Ir_{0.5}$	$Au_{0.7}Ir_{0.3}$	Ir	Pt	Au
Size (nm)	5.5±1.4	7.2±1.7	9.9±2.3	1.4±0.4	5.7±0.7	7.1±1.2

7. Elemental analysis of the particle surface of $Au_{0.5}Ir_{0.5}$ NP

The metal composition of the surface of $Au_{0.5}Ir_{0.5}$ NP was investigated by EDX point analysis using a JEM-200CF. The average composition x in Au_xIr_{1-x} was calculated to be 0.43 ± 0.13.

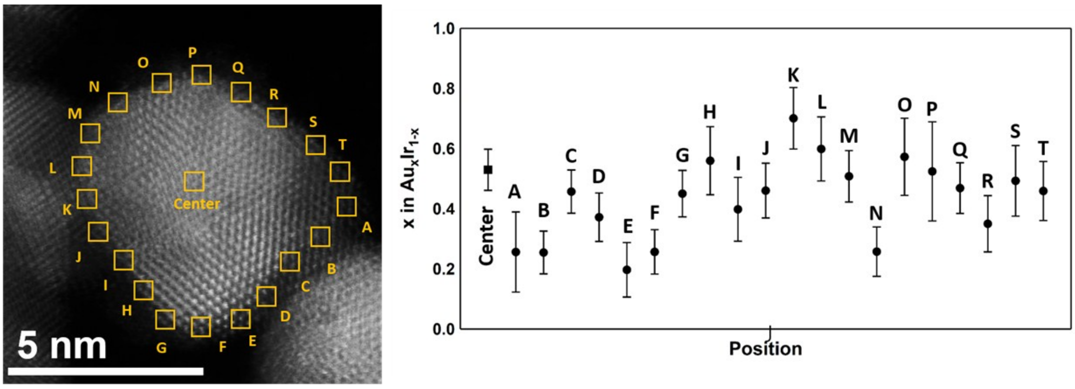

Figure S10 HAADF-STEM image $Au_{0.5}Ir_{0.5}$ NP and the results of EDX point analysis. The analysed positions were marked in the HAADF-STEM image.

8. STEM analysis of $Au_{0.2}Ir_{0.8}$ and $Au_{0.7}Ir_{0.3}$ NPs

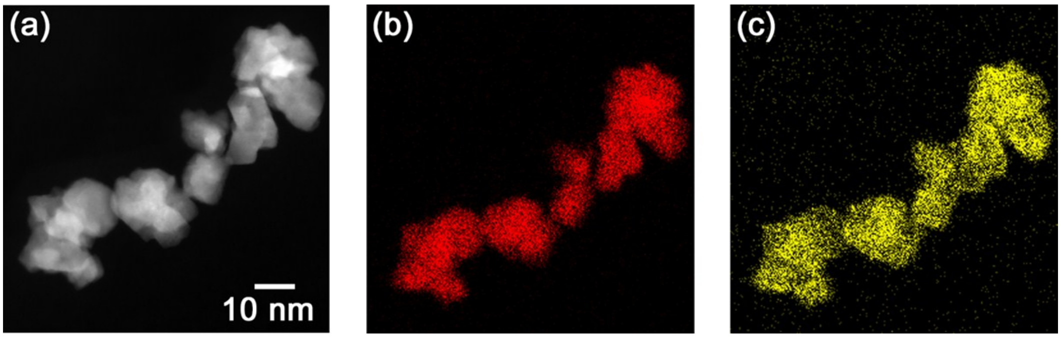

Figure S11 Scanning transmission electron microscopy of $Au_{0.2}Ir_{0.8}$ NPs. **a**, a HAADF-STEM image. **b, c**, Au-M and Ir-M STEM−EDX maps (red, Au; yellow, Ir).

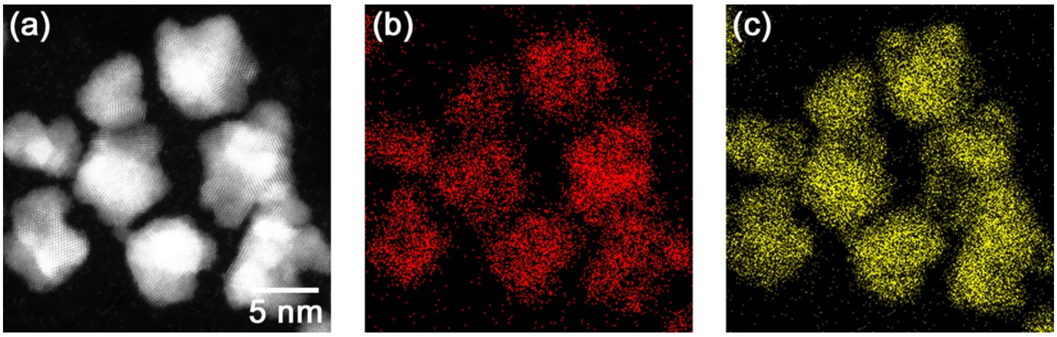

Figure S12 Scanning transmission electron microscopy of $Au_{0.7}Ir_{0.3}$ NPs. **a**, a HAADF-STEM image. **b, c**, Au-M and Ir-M STEM−EDX maps (red, Au; yellow, Ir).

9. Elemental analysis of the prepared Au_xIr_{1-x} and Pt catalysts

The loading amount of metals on the carbon support was characterized by CHN elemental analysis. The average value was calculated as shown in Table S12.

Table S12. The loading amount of metals on the carbon support

	$Au_{0.2}Ir_{0.8}$	$Au_{0.5}Ir_{0.5}$	$Au_{0.7}Ir_{0.3}$	Ir	Pt	Au
wt%	21.8	24.0	23.4	23.6	25.4	21.5

10. CV curves of each catalyst

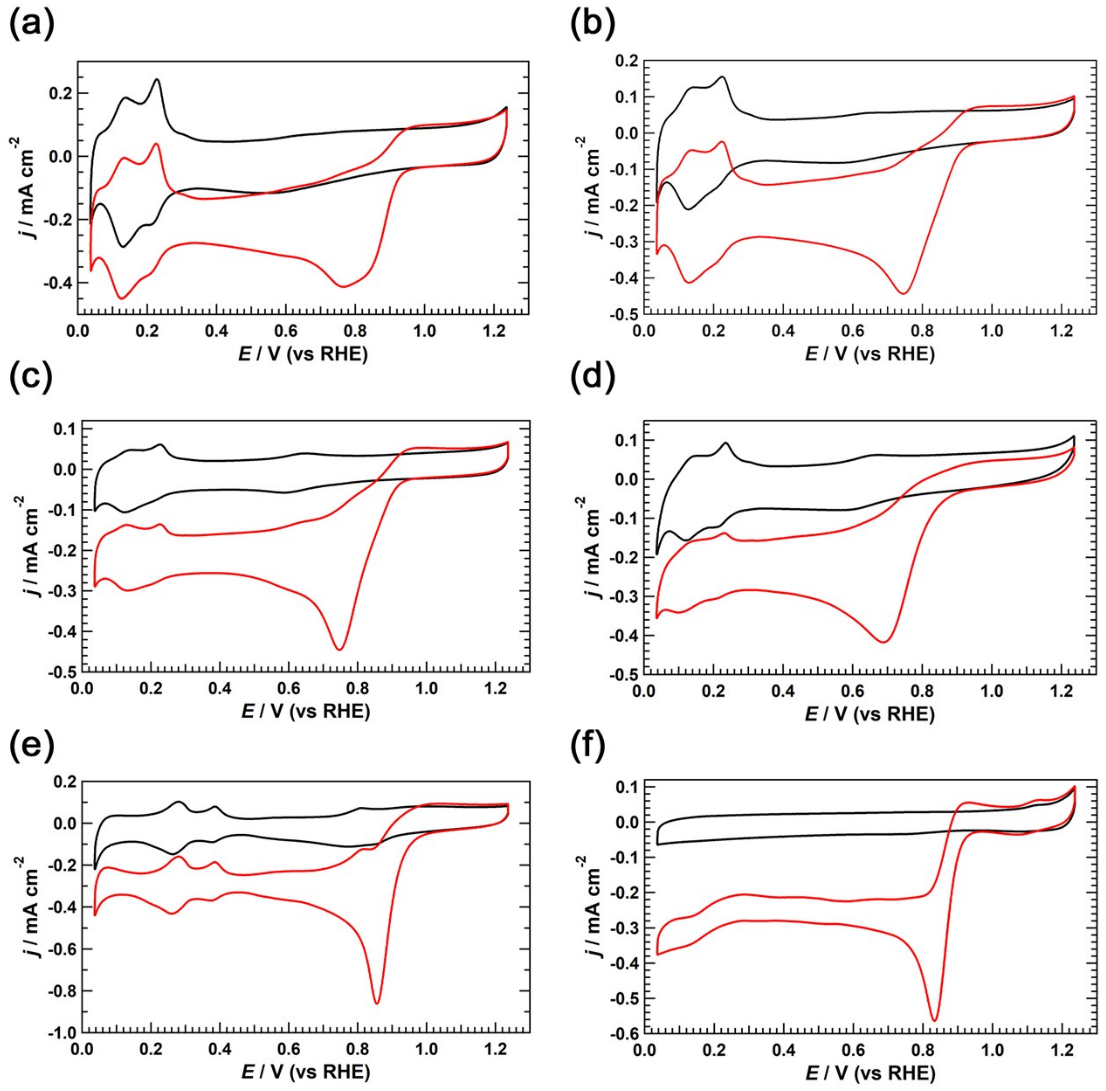

Figure S13 Cyclic voltammograms of (a) $Au_{0.2}Ir_{0.8}$, (b) $Au_{0.5}Ir_{0.5}$, (c) $Au_{0.7}Ir_{0.3}$, (d) Ir, (e) Pt, and (f) Au catalysts. Black and red curves indicate the CV obtained in Ar and O_2 saturated 1.0 M NaOH solutions, respectively. The scan rate was 20 mV/sec.

11. Electrochemical oxygen-reduction activities in an acidic solution

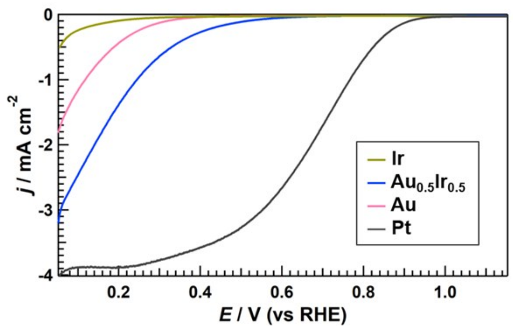

Figure S14 RDE polarization curves of $Au_{0.5}Ir_{0.5}$ and monometallic catalysts at 1600 rpm in an O_2-saturated 0.05 M H_2SO_4 aqueous solution. The catalytic tests were performed in the same procedure as in 1.0 M NaOH aqueous solution case.

12. accelerated durability test of $Au_{0.5}Ir_{0.5}$ catalyst

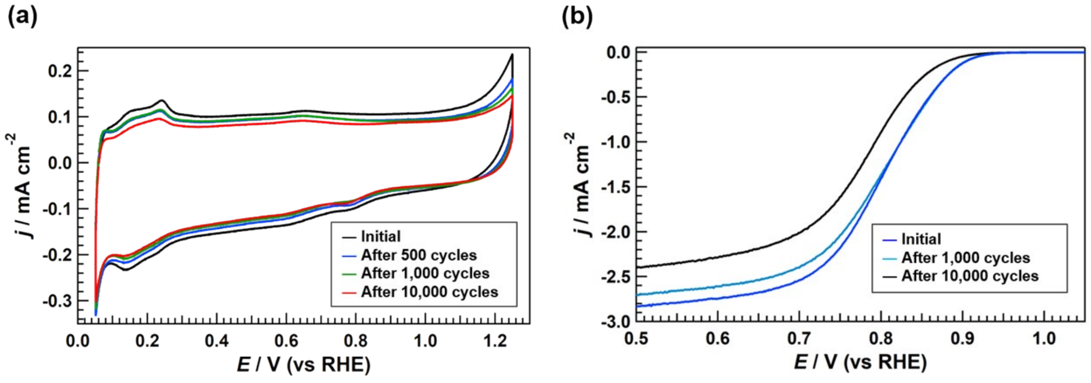

Figure S15 (a) Cyclic voltammograms of $Au_{0.5}Ir_{0.5}$ in an Ar-saturated 1.0 M NaOH aqueous solution before and after ADT. The durability test was carried out for the same sample at room temperature in an O_2-saturated 1.0 M NaOH aqueous solution with the cyclic potential sweeping between 0.65 and 0.95 V (vs RHE) at a sweep rate 100 mV/s. (b) RDE polarization curves of $Au_{0.5}Ir_{0.5}$ before and after ADT at 1600 rpm in an O_2-saturated 1.0 M NaOH aqueous solution.

Table S13. The H_{upd} peak area (Q_H) (calculated from 0.1 to 0.4 V (vs RHE)) of $Au_{0.5}Ir_{0.5}$ catalyst before and after ADT test

	Initial	After 500 cycles	After 1,000 cycles	After 10,000 cycles
Q_H (C)	1.07×10^{-4}	1.03×10^{-4} (96.7%)	1.01×10^{-4} (94.2%)	9.84×10^{-4} (92.1%)
